# Heart rhythm characterization through induced physiological variables

**DOI:** 10.1038/s41598-017-04998-7

**Published:** 2017-07-11

**Authors:** Jean-François Pons, Zouhair Haddi, Jean-Claude Deharo, Ahmed Charaï, Rachid Bouchakour, Mustapha Ouladsine, Stéphane Delliaux

**Affiliations:** 1 Aix Marseille Univ., Univ. Toulon, CNRS, IM2NP, Marseille, France; 2 Aix Marseille Univ., Univ. Toulon, CNRS, ENSAM, LSIS, Marseille, France; 3 Aix Marseille Univ., IRBA, DS-ACI, Marseille, France; 4 APHM, Hôpital La Timone, Service de Cardiologie du pôle cardiovasculaire et thoracique, Marseille, France; 5 APHM, Hôpital Nord, Service des Explorations Fonctionnelles Respiratoires, Pôle cardiovasculaire, Marseille, France

## Abstract

Atrial fibrillation remains a major cause of morbi-mortality, making mass screening desirable and leading industry to actively develop devices devoted to automatic AF detection. Because there is a tendency toward mobile devices, there is a need for an accurate, rapid method for studying short inter-beat interval time series for real-time automatic medical monitoring. We report a new methodology to efficiently select highly discriminative variables between physiological states, here a normal sinus rhythm or atrial fibrillation. We generate induced variables using the first ten time derivatives of an RR interval time series and formally express a new multivariate metric quantifying their discriminative power to drive state variable selection. When combined with a simple classifier, this new methodology results in 99.9% classification accuracy for 1-min RR interval time series (n = 7,400), with heart rate accelerations and jerks being the most discriminant variables. We show that the RR interval time series can be drastically reduced from 60 s to 3 s, with a classification accuracy of 95.0%. We show that heart rhythm characterization is facilitated by induced variables using time derivatives, which is a generic methodology that is particularly suitable to real-time medical monitoring.

## Introduction

Despite constant improvements in the management of atrial fibrillation (AF), this arrhythmia remains a major cause of mortality and severe morbidity. The diagnosis of AF is key in primary and secondary prevention of cardiovascular mortality and morbidity. As it is frequently asymptomatic, AF is often unrecognized. Implementation of systematic AF screening programmes is strongly recommended, particularly in at-risk populations^1^. In recent years, rapid technological development has led to many new commercially available tools intended to automatically detect arrhythmias, from smartphone applications to smart watches and noncontact ECG methods^1–5^. Most AF detection methods are based on inter-beat interval time series analysis.

Among the existing approaches dedicated to automatic AF detection, attention has been devoted to RR interval time series rather than atrial activity (p-wave detection). The high-intensity noise often present in ECGs may overlap with the p-wave, limiting AF detection. Various univariate and multivariate techniques have been applied to RR interval analysis. For instance, K. Tateno and L. Glass applied the coefficient of variation and density histograms of the RR interval and ΔRR interval as templates for AF detection^6^. The differences between the standard histograms (benchmark) and a test record were quantitatively evaluated using the Kolmogorov-Smirnov (KS) test and p-value statistical significance. By comparing the test records, they demonstrated that the density histogram of the ΔRR interval is more accurate for most subjects (average sensitivity of 93.2% and average specificity of 96.7%) than RR interval density histograms. However, short-duration AF leads to a low specificity. S. Dash *et al*. investigated optimal thresholds of the Turning Points Ratio in combination with the Root Mean Square of Successive RR interval Differences and the Shannon Entropy^7^. Ectopic beats were suppressed before parameter calculation. The presence of AF using this method was considered if given conditions based on the obtained thresholds were satisfied. The calculated sensitivity and specificity were 94.4% and 95.1%, respectively, for the PhysioNet afdb database and 90.2% and 91.2%, respectively, for the MIT-BIH Arrhythmia Database.

X. Zhou and co-workers detected AF based on symbolic dynamics of ΔRR and Shannon entropy^8^. The best performance in terms of sensitivity and specificity was 96.72% and 95.07%, respectively, for the training set (Long-Term AF Database). For a test dataset containing AF and normal sinus rhythm (MIT-BIH afdb), the sensitivity and specificity were 96.89% and 98.25%, respectively. However, the authors noted that sporadic AF episodes of relatively short duration (e.g., ten seconds) might incur false negative detection and that this may be a potential limitation.

Although the findings are acceptable in terms of sensitivity and specificity, univariate analysis often refers to a unique feature to make decisions. In contrast, multivariate analysis attempts to couple several variables with confirmed pattern recognition methods, which is beneficial for complex problems such as automatic AF detection. J. Park *et al*. exploited three features extracted from heart rate variability in Poincaré plots as inputs to K-means clustering methods and Support Vector Machines with radial basis function kernel^9^. The performance of the SVM was evaluated using a leave-one-out cross-validation method on the Paroxysmal Atrial Fibrillation Prediction Challenge Database^10^ and the Atrial Fibrillation Termination Challenge Database^11^. The mean sensitivity and specificity were 91.4% and 92.9%, respectively. M. Carrara *et al*. compared three multivariate techniques, namely, logistic regression, K-nearest neighbours, and random forests, to distinguish between AF, normal sinus rhythm (NSR) and sinus rhythm (SR) with frequent premature beats^12^. The random forests analysis applied to five features (mean of RR interval segment, standard deviation, coefficient of sample entropy, local dynamics score and detrended fluctuation analysis) had positive predictive values of 97, 98 and 90% for AF, NSR, and SR with premature beats, respectively. However, this classifier assumed atrial flutter to be the same as AF. R. Acharya *et al*. classified nine cardiac abnormalities including AF and NSR using multilayer perceptron artificial neural networks and the spectral parameters of the autoregressive moving average (ARMA) model as relevant features^13^. The classifier achieved 83.33% correct classification of the studied diseases.

**Figure 1:**
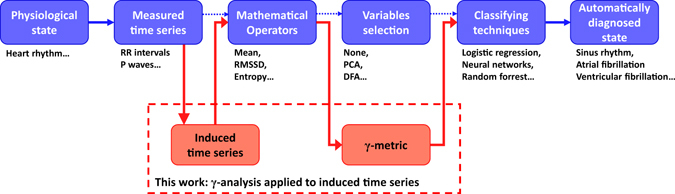
Classic physiology automatic decision flow chart process: Standard steps of the usual approach to physiological state automatic classification are shown as blue boxes. Only some examples are given for each step according to the focused arrhythmia problem. The contribution of this work is highlighted in the dashed red box and consists of applying the γ-analysis, a simple method we developed to easily screen many potential discriminant variables through calculation of the γ-metric for an extended set of state variables generated by the calculation of induced time series (time derivative of a measured RR interval time series). The red boxes and red arrows show that the positioning of our proposal is complementary to the typical steps of the automatic decision flow chart process in physiology.

As in univariate analysis, these RR interval-based variables (and others) do not yield results with 100% accuracy. All univariate and multivariate analyses required 100- to 200-beat sequences and most authors noted that shorter sequences would lead to dramatic loss of accuracy. Because most reported studies focused on variables extracted from a unique source (i.e., RR interval time series), we believe that time-domain, frequency-domain, and non-linear variables^14–17^ do not contain exhaustive information of the underlying heart rhythm. By analogy with kinematics, which states that a dynamical state is better characterized by knowing its position, displacement, speed, and acceleration rather than a single position, we hypothesize that the use of induced physiological variables (i.e., time derivatives of RR interval time series) can extend the set to include additional information sources and enable rapid and efficient clinical decision support. This paper presents a novel methodology to improve physiological and clinical state characterization. We focus on the specific example of heart rhythm characterization. However, it should be noted that we do not propose a new classifier tool to challenge those mentioned above. Rather, we suggest how to generate, select and provide new input variables to existing classifier tools to study induced time series (time-derivative RR interval time series) derived from observed time series that are RR interval time series. The position of our work is illustrated in Fig. 1, which highlights how our methodology increases the field of view of a dynamic physiological phenomenon and the information used to characterize it. This should counteract the lack of observation time in daily medical practice that drastically limits the exploitation of physiological time series^18, 19^ as advances in technology and computerized calculation enable clinicians to work with time series at the bedside^20–24^ through new real-time mobile devices under development.

## Results

### The *γ*-metric quantifies heart rhythm discriminability

**Figure 2:**
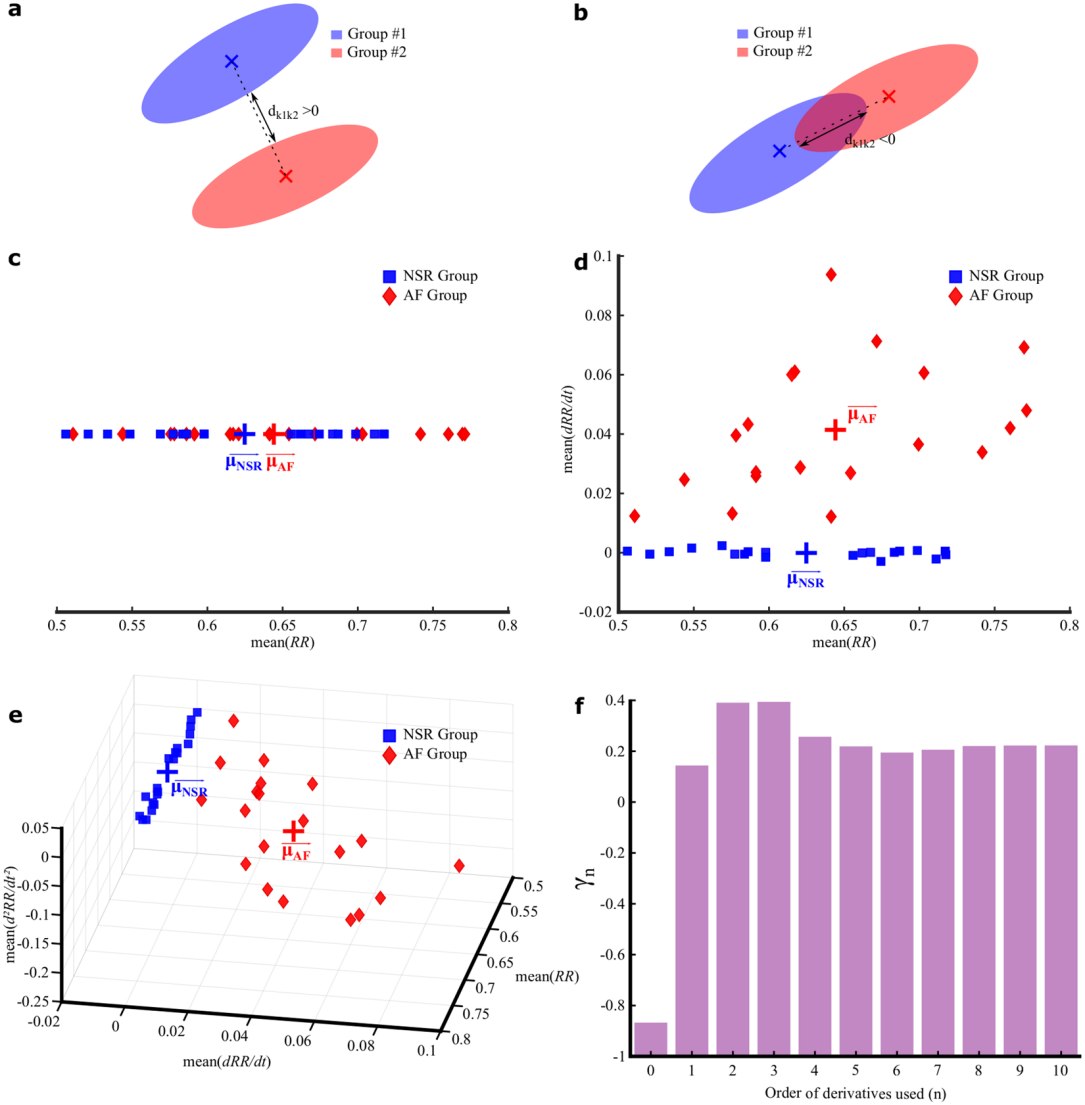
Illustrative example of group discrimination using the γ-metric. (**a**,**b**) Generic 2-dimensional illustrations of the distance d_k1k2_ used in the γ-analysis to discriminate two groups; d_k1k2_ is positive for non-intersecting groups (**a**) and negative for intersecting groups (**b**). (**c**) Unidimensional representation of the Normal Sinus Rhythm (NSR, N = 20) group and the Atrial Fibrillation (AF, N = 20) group in terms of the means of 1-min RR interval time series. µ_NSR_ and µ_AF_ represent the centres of mass of the NSR and AF groups, respectively. (**d**) Bidimensional representation of the NSR group and the AF group in terms of the mean RR interval and its first time derivative. (**e**) Tridimensional representation of the NSR group and the AF group in terms of the mean RR interval and its first and second time derivatives. (**f**) Evolution of the γ-metric value when the averages of the first n derivatives are jointly used as state variables to characterize heart rhythms as NSR or AF from the dataset shown in (**c**–**e**).

An illustrative simple example of group discrimination using the algebraic geometric distance expressed by the γ-metric is shown in Fig. 2. The *γ*-metric value can be positive (separate groups along the mean-mean axis, Fig. 2a) or negative (overlapping groups along the mean-mean axis, Fig. 2b). The higher the *γ*-metric value, the easier the group discrimination is (Fig. 2c–f). When applied to heart rhythm discrimination using an increasing number of variables to describe NSR and AF 1-min RR interval time series, the geometric distance in the selected state space between the NSR and AF groups and the corresponding *γ*-metric increase when using derivatives up to order three, as depicted in Fig. 2c–f. Inclusion of higher orders of derivation leads to a decrease in the *γ*-metric by almost 50%. The *γ*-metric can be used to rank the discriminant power of the variables that describe NSR and AF groups and allows for targeting the most relevant ones.

### Derived time series improve heart rhythm characterization

**Figure 3:**
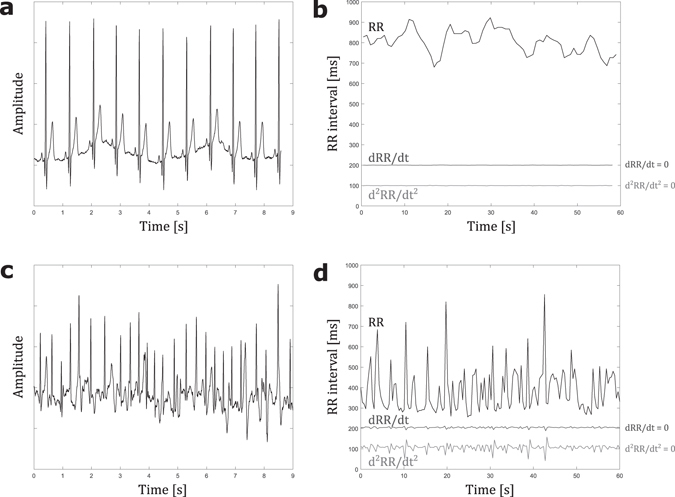
Illustrative example of ECG recording and time series generation. (**a**) Short fraction of ECG recording n° 16773 extracted from PhysioNet MIT-BIH nsrdb from t = 0 s to t = 9 s, showing a normal sinus rhythm. (**b**) One-minute RR interval time series (black tachograms) generated after R peak detection with a homemade algorithm of n° 16773 ECG recording (**a**) from t = 0 s to t = 60 s. Induced time series dRR/dt and d^2^RR/dt^2^ (first and second time derivatives time series computed from the initially generated RR interval time series) are also plotted in dark and light grey, respectively. (**c**) Short fraction of ECG recording n° 04126 extracted from PhysioNet MIT-BIH afdb from t = 97 s to t = 106 s, showing an atrial fibrillation. (**d**) One-minute RR interval time series (black tachograms) generated after R peak detection with a homemade algorithm of n° 04126 ECG recording (**c**) from t = 97 s to t = 157 s. Induced time series dRR/dt and d^2^RR/dt^2^ (first and second time derivatives time series computed from the initially generated RR interval time series) are also plotted in dark and light grey, respectively.

**Figure 4:**
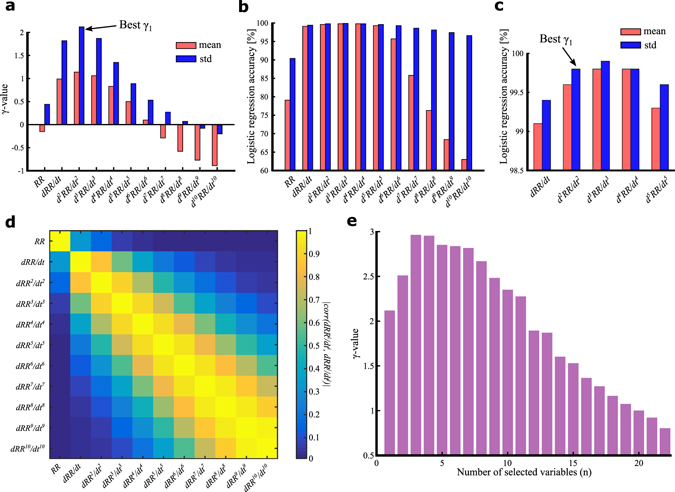
Optimizing state characterization using the γ-metric. (**a**) γ-values for the mean and standard deviation operators applied to 1-min RR interval time series and their first ten derivatives for Normal Sinus Rhythm (NSR, N = 4,500) and Atrial Fibrillation (AF, N = 2,900) states. (**b**) Percentage accuracies for the subsequent univariate logistic regression classification of 7,400 heart rhythm states with respect to the operators (mean and standard deviation) and time derivatives used to characterize the 1-min RR interval time series. (**c**) Zoomed-in view of the cases with the highest accuracy depicted in (**b**). The best γ_1_ selection is noted for comparison with the subsequent best classification accuracy. (**d**) Correlations between the 11 time series used to characterize the heart rhythm states (the RR interval time series and its first ten derivatives) expressed as a matrix of the absolute values of the Pearson correlation coefficients. (**e**) Highest γ-values for all n-variable combinations of the generated variables (means and standard deviations of the RR interval time series and each of the first ten derivatives). This multivariate γ-analysis indicates that the maximum discriminatory power (highest γ-value) is achieved with three variables.

Illustrative examples of NSR and AF ECGs are shown in Fig. 3 with their respective RR interval time series (tachograms) and their first- and second-order time-derived time series. Here, the *γ*-metric is applied for heart rhythm classification as NSR or AF. Table 1 shows the mean ± standard deviation for the mean and standard deviation operators applied to each studied time series for two groups defined according to their medical classification as NSR or AF. All observed differences were significant (p < 0.001). The corresponding *γ*-values are also reported. The *γ*-metric shows that derived time series can lead to improved NSR/AF group discrimination and that, for a given derivative order (from 0 to 10), the application of the standard deviation operator to a time series offers more discriminative power (a higher *γ*-metric value) than the mean operator. This finding is emphasized in Fig. 4a, which shows the *γ*-values for the mean and standard deviation operators applied to each time series (derivatives up to the 10^th^ order of the initial tachogram) for the specific NSR/AF classification problem of interest. In addition, Fig. 4a shows that, starting from the initial RR interval tachogram, the *γ*-metric increases as the derivative order increases up to a certain value (the 2^nd^ order, in this case) and then subsequently decreases. The best *γ*-value was obtained by applying the standard deviation operator to the second-order derivative of the RR time series, which can be understood as a metric that highlights the heart rhythm dynamics (heart rate jerk). This indicates that if only one variable is to be used to classify a new sample as NSR or AF, better classification should be achieved using the standard deviation of the second-order derivative. To this end, Fig. 4b and c show the percentage accuracies obtained with an unregularized logistic regression classifier trained with each of the 22 variables. The variable selected through the best *γ*_1_ results in one of the best discriminative variable selections after an unregularized logistic regression classifier is used. In this example, the best classification is obtained with the standard deviation of the third-order time derivative, but the classification accuracy difference compared to that obtained with the best *γ*_1_ (0.1%) is not statistically significant (only 1 misclassified sample in the validation set).

**Table 1:** Application of the γ-metric for heart rhythm classification.

Derived variable	NSR Group	AF Group	*γ*_*NSR*/*AF*_
Mean			
RR	0.80 ± 0.15	0.61 ± 0.08***	−0.15
dRR/dt	0.00 ± 0.00	−0.04 ± 0.02***	0.99
d^2^RR/dt^2^	0.00 ± 0.00	0.12 ± 0.05***	1.14
d^3^RR/dt^3^	0.00 ± 0.00	−0.32 ± 0.15***	1.06
d^4^RR/dt^4^	0.00 ± 0.01	0.75 ± 0.40***	0.83
d^5^RR/dt^5^	−0.01 ± 0.02	−1.67 ± 1.09***	0.50
d^6^RR/dt^6^	0.01 ± 0.06	3.69 ± 3.28***	0.10
d^7^RR/dt^7^	−0.02 ± 0.20	−7.99 ± 11.01***	−0.29
d^8^RR/dt^8^	0.05 ± 0.75	17.09 ± 40.15***	−0.58
d^9^RR/dt^9^	−0.10 ± 2.38	−37.09 ± 158.82***	−0.77
d^10^RR/dt^10^	0.39 ± 11.00	72.63 ± 655.95***	−0.89
Standard deviation			
RR	0.04 ± 0.02	0.12 ± 0.03***	0.44
dRR/dt	0.03 ± 0.02	0.27 ± 0.07***	1.82
d^2^RR/dt^2^	0.06 ± 0.03	0.80 ± 0.20***	2.12
d^3^RR/dt^3^	0.14 ± 0.10	2.58 ± 0.75***	1.87
d^4^RR/dt^4^	0.37 ± 0.33	8.83 ± 3.27***	1.35
d^5^RR/dt^5^	1.01 ± 1.19	31.77 ± 15.13***	0.89
d^6^RR/dt^6^	2.92 ± 4.55	118.75 ± 71.15***	0.53
d^7^RR/dt^7^	8.91 ± 18.03	457.43 ± 335.68***	0.27
d^8^RR/dt^8^	28.27 ± 73.76	1806.84 ± 1586.20***	0.07
d^9^RR/dt^9^	93.13 ± 309.85	7290.95 ± 7516.51***	−0.08
d^10^RR/dt^10^	317.51 ± 1330.37	29965.71 ± 35747.55***	−0.20

Mean ± standard deviation values for the mean and standard deviation operators applied to the first ten time derivatives of the initial RR time series for comparison of the Normal Sinus Rhythm (NSR, N = 4,500) group with the Atrial Fibrillation (AF, N = 2,900) group, ***p-value < 0.001 by Student’s t-test and the univariate *γ*_*NSR*/*AF*_ value that represents the surface-to-surface algebraic distance between the two 1-dimensional ellipsoids (segments) that correspond to the NSR and AF groups.

### Multivariate *γ*-analysis optimizes heart rhythm discrimination and classification

The correlations between the observed and induced variables are shown in Fig. 4d. Derivatives of the initial time series may be useful in a classification problem because these derivatives are not fully correlated with the initial time series (see the first row or the first column of the figure). As the derivative order increases, the correlations with higher-order derivatives become stronger (increasing along the diagonal towards the bottom right of the figure). However, even with a high-order derivative, it is possible to find a derivative of another order that is only partially correlated with it. Thus, using induced variables to increase the dimensionality of the state space may be beneficial for characterization because uncorrelated induced variables may be interpreted as variables that extract different aspects of information contained in the initially observed variable.

Consequently, more than one variable may be used to address classification problems, and *γ*-analysis can be used to rank induced variables and their combinations according to their respective discriminant power. The *γ*-value was calculated for each combination of *i* ∈ {1..22} variables, and the largest *γ*-value obtained for each number of selected variables *i* is presented in Fig. 4e. This multivariate γ-analysis indicates that the maximum discriminatory power (highest γ-value) is achieved with three variables. Adding more variables does not further increase the *γ*-value; this finding indicates the limited usefulness of such additional variables for this classification problem. The details of the selected variables are summarized in Table 2, where the number of variables is limited to 10 for brevity as three variables are sufficient for classification. The highest *γ*_1_, *γ*_2_ and *γ*_3_ values were obtained with the single variable $\{\sigma (\frac{{d}^{2}RR}{d{t}^{2}})\,\}$, the pair of variables $\{\sigma (\frac{{d}^{2}RR}{d{t}^{2}}),\,mean(\frac{{d}^{2}RR}{d{t}^{2}})\,\}$ and the triplet of variables $\{\sigma (\frac{{d}^{2}RR}{d{t}^{2}}),\,mean(\frac{dRR}{dt}),\,\sigma (\frac{dRR}{dt})\,\}$, respectively, where *σ* denotes the standard deviation operator. A complete study of all pairs of variables shows that classification accuracy from 69.6% to 99.9% is achieved and that the pair of variables selected by the *γ*-analysis is among the most discriminative, reaching 99.8%.

**Table 2:** Variables selected through γ-analysis.

n-variables model/#variables	1	2	3	4	5	6	7	8	9	10
*mean* (*RR*)									✓	✓
*σ* (*RR*)					✓	✓	✓	✓	✓	✓
$mean(\frac{dRR}{dt})$			✓	✓	✓	✓	✓	✓	✓	✓
$\sigma (\frac{dRR}{dt})$			✓	✓	✓	✓	✓	✓	✓	✓
$mean(\frac{{d}^{2}RR}{d{t}^{2}})$		✓		✓	✓	✓	✓	✓	✓	✓
$\sigma (\frac{{d}^{2}RR}{d{t}^{2}})$	✓	✓	✓	✓	✓	✓	✓	✓	✓	✓
$mean(\frac{{d}^{3}RR}{d{t}^{3}})$							✓	✓	✓	✓
$\sigma (\frac{{d}^{3}RR}{d{t}^{3}})$						✓	✓	✓	✓	✓
$mean(\frac{{d}^{4}RR}{d{t}^{4}})$								✓	✓	✓
$\sigma (\frac{{d}^{4}RR}{d{t}^{4}})$										✓

For a given number n (1–10) of selected variables used in a classifier model, the table shows the combinations of variables that yield the highest γ-values when the mean and standard deviation operators are applied to the RR interval time series (N = 7,400) and their four first time derivatives.

### Applicability to short time series

**Figure 5:**
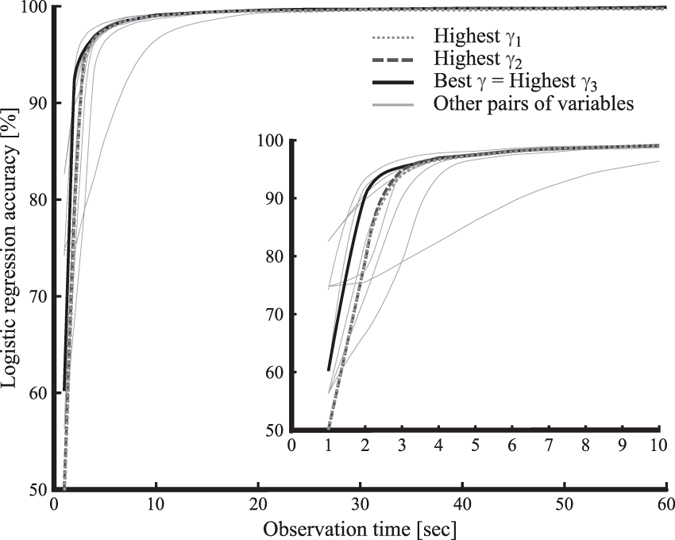
Evolution of the classification accuracy with decreasing observation time: The accuracies of multivariate logistic regressions in discriminating the Normal Sinus Rhythm (NSR, N = 900) and Atrial Fibrillation (AF, N = 580) states for RR intervals measured over observation times from 1 to 60 s. The dotted grey line represents the fitted accuracy curve for the best single variable selected through γ-analysis (best γ_1_), the dark dashed grey line represents the fitted accuracy curve for the best pair of variables selected through γ-analysis (best γ_2_), the black line represents the fitted accuracy curve for the best triplet of variables selected through γ-analysis (best γ_3_), and the thin grey lines represent other combinations of two variables.

Because introducing induced state variables to characterize the derived time series was shown to enable better use of the available information than that achieved using the original data, this approach was used to reduce the observation time required to classify the rhythm state. The evolution of the classification accuracy with decreasing observation time (shorter time series) is depicted in Fig. 5. In this figure, the selected *γ*_1_, *γ*_2_ and *γ*_3_ correspond to the highest *γ*-value when 1, 2 and 3 variables, respectively, are used to describe the state space according to Table 2. In these cases, Fig. 5 shows that, even for observations as short as 5s, the classification accuracy of logistic regression for this database is higher than 95%, allowing for faster classification.

### Robustness of the *γ*-analysis applied to induced RR interval time series

**Figure 6:**
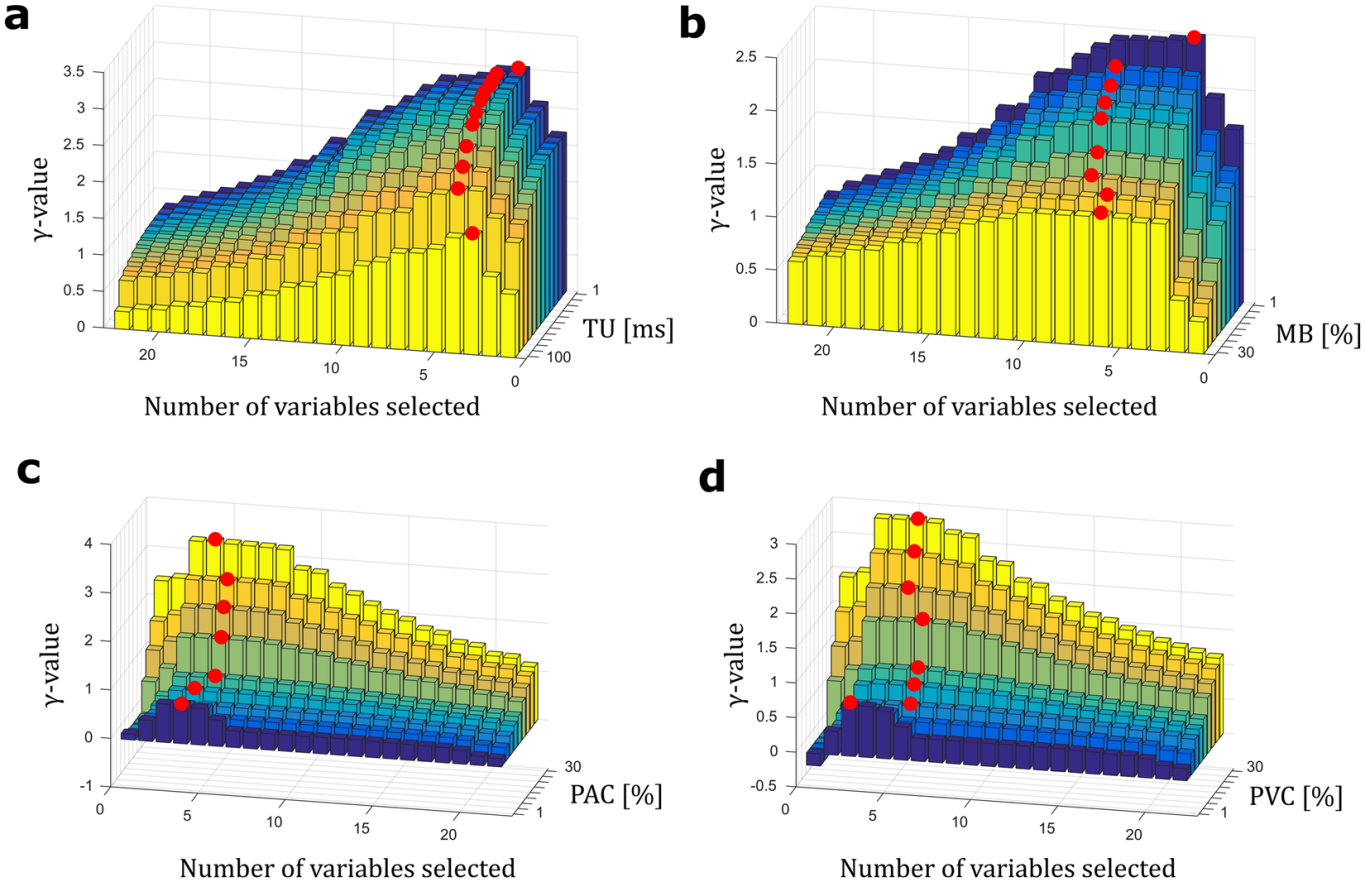
Robustness of the γ-metric to noisy data. (**a**) γ-value plotted as a function of the time uncertainty (TU, ms) applied to each R peak detection of the studied RR interval time series for each of the 1- to 22-variable models. (**b**) γ-value plotted as a function of the missing beats (MB, %) percentage applied to each RR interval time series for each of the 1- to 22-variable models. (**c**) γ-value plotted as a function of the premature atrial contraction (PAC, %) percentage applied to each RR interval time series for each of the 1- to 22-variable models. (**d**) γ-value plotted as a function of the premature ventricular contraction (PAC, %) percentage applied to each RR interval time series for each of the 1- to 22-variable models. In a, b, c, and d, increasing noise is indicated from dark blue (light noise) to bright yellow (strong noise). For each noise step, the red dot highlights the best n-variable model, i.e., the number of variables included in the model that has the highest γ-value. The analyses were performed on 7,400 1-min RR interval time series (NSR = 4,500, AF = 2,900).

Data acquired through real-time embedded devices are often corrupted by different sources of perturbations such as timing uncertainty on the real peak positions given by peak detector algorithms applied to noisy data or missing data points. Sinus rhythm labelling of heart rhythm does not exclude the presence of premature contractions, which is not necessarily pathological but is often a source of automatic misclassification. Therefore, to address the robustness of the proposed technique, the variable selection was conducted on a corrupted database. The perturbations used to characterize the robustness of the proposed techniques are the peak detection timing uncertainty (TU), the percentage of missing beats (MB), the percentage of premature atrial contractions (PAC) and the percentage of premature ventricular contractions (PVC). The models used to describe these perturbations are detailed in the methods section. Figure 6 represents how the *γ*-value evolves when the TU, MB, PAC and PVC perturbations are used to corrupt the rhythm states for separation. The *γ*-analysis adapts the selected variables that are more prone to discriminate the two groups. For brevity, the variables selected are not detailed here but the red dots (representing the highest *γ*-value for the fixed amount of perturbation) show that the number of variables needed to discriminate the two AF and NSR groups can increase. This is especially visible in Fig. 6b to d, where the number of variables selected by the *γ*-analysis is between 4 and 7.

**Figure 7:**
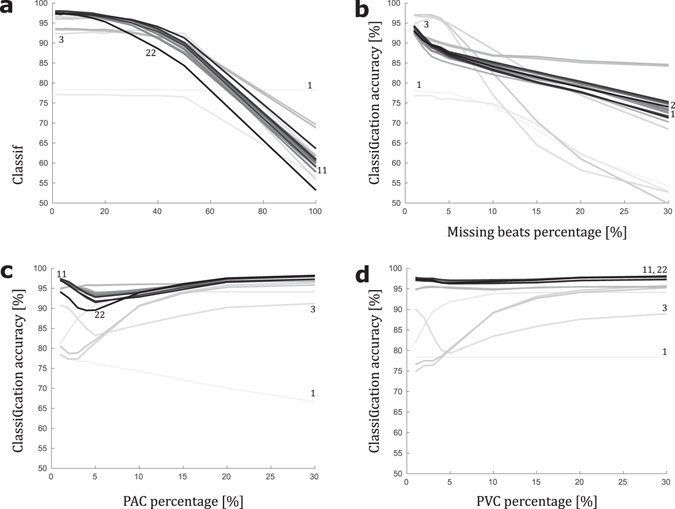
Robustness of the γ-analysis to noisy data. Classification accuracy of multivariate logistic regression in discriminating the Normal Sinus Rhythm (NSR, N = 4,500) and Atrial Fibrillation (AF, N = 2,900) states for 1-min RR interval time series, combining 1 to 22 input variables according to the 1 to 22 n-tuples with the highest γ-value identified among the $$\sum _{k=1}^{22}(\begin{array}{c}22\\ k\end{array})$$ possible combinations in the preliminary γ-analysis selection plotted against (**a**) timing uncertainty applied to each R peak detection, (**b**) missing beats percentage applied to each RR interval time series used, (**c**) premature atrial contractions (PAC) percentage applied to each RR interval time series used, (**d**) premature ventricular contractions (PVC) percentage applied to each RR interval time series used. In (**a**–**d**), all models are colour-coded from light grey to black according to the number of included variables (from 1 to 22). Extrema are labelled, 1-variable and 22-variable models, as well as the best model (3 variables) defined by the γ-analysis when learning was performed on a noiseless dataset and the best model (11 variables) γ-analysis when learning was performed on pooled noisy datasets.

Because the variable selection in the *γ*-analysis depends on the shapes of the groups to discriminate, a generalized variable selection was attempted with the initial AF and NSR databases to which missing beats, PAC and PVC were added. This mixed NSR database is detailed in the methods section. With these two groups selected, we applied an exhaustive *γ*-analysis to select 1 to 22 variables for these groups. All 22 groups of variables were used as inputs for an unregularized logistic regression classifier. The accuracies obtained with this classifier are shown in Fig. 7 for the four sources of perturbation and for the selection of variables in the *γ*-analysis. The resulting best *γ* variable combination was a group of 11 variables. In this figure, the accuracy of the unregularized logistic regression with the best *γ*_1_, the best *γ*_11_ and the *γ*_22_ variable combination is highlighted. The new best *γ*_3_ variable combination is highlighted to compare with the number of variables selected on noiseless databases. The remainder of the curves are not numerated, but the darkness of the curves gradually increases with the number of variables selected (lightest = 1 variable, darkest = 22 variables). Figure 7 shows that despite the introduced perturbations, the *γ*-analysis allows for selection of a reduced number of variables associated with high classifier accuracies. Notably, as illustrated in Fig. 7a to c, using more variables does not imply a better accuracy, primarily due to overfitting of the training database. Thus, the *γ*-analysis helps to select a reduced set of variables to prevent this phenomenon.

**Figure 8:**
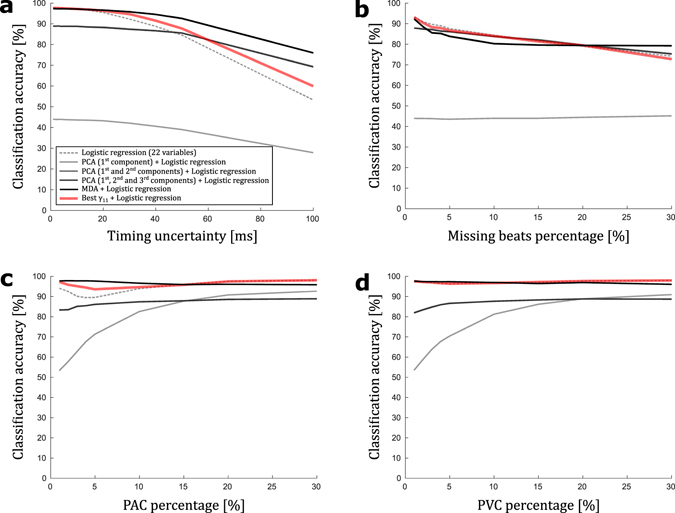
Impact of variable selection technique on classification accuracy. Classification accuracy of multivariate logistic regression in discriminating the Normal Sinus Rhythm (NSR, N = 4,500) and Atrial Fibrillation (AF, N = 2,900) states for 1-min RR interval time series applied to noisy datasets plotted against (**a**) timing uncertainty applied to each R peak detection, (**b**) missing beats percentage applied to each RR interval time series used, (**c**) premature atrial contractions (PAC) percentage applied to each RR interval time series used, (**d**) premature ventricular contractions (PVC) percentage applied to each RR interval time series used. In (**a**–**d**), the management of input variables candidates consisted of no preliminary selection (22 input variables, dotted line line), selection of the first component (1 input variable that is a combination of the 22 available variables, light grey), selection of the 2 first components (2 input variables that are a combination of the 22 available variables, medium grey), and selection of the 3 first components (3 input variables that are a combination of the 22 available variables, dark grey) of a principal component analysis (PCA), selection of the factorial axis of the multiple discriminant analysis (1 input variable that is a combination of the 22 available variables, black), and selection of the best γ-value (11 input variables among the 22 available variables, red).

The *γ*-analysis was compared to other techniques used to select variables before use as inputs to a classifier. We compared the best *γ*_11_ selection to no selection at all, to the selection of the first one, first two and first three components of the Principal Component Analysis (PCA) and to the variable selected by the Multiple Discriminant Analysis (MDA). For the PCA, only the first three components were considered because they explained 99.85%, 99.97% and 100% of the variance. The comparison of techniques for the selection of variables and the *γ*-analysis was done by comparing the consequent logistic regression accuracies. The results are depicted in Fig. 8 for the abovementioned types of perturbations. The results of the classification conducted with variables selected by the *γ*-analysis appear to be generally similar to those conducted with other well-known techniques. When compared to the best classification results (obtained using the MDA), Fig. 8 shows that the reduced set of variables obtained from the *γ*-analysis results in similar classification accuracy without the need to process and store all 22 variables used by the hyperplane described by the MDA. Notably, with 99.85% of the input set variance explained by the first component, one could use the first PCA component only and obtain very bad classification results.

## Discussion

In this paper, we developed a new approach for characterizing physiological states to solve medical diagnostic challenges and tested it on 7,600 1-min heart rate time series. We highlighted the benefits of automatic AF detection using induced variables as accelerations and jerks calculated from the original instantaneous heart rate. We developed a methodology based on the proposed *γ*-metric to ensure that the selected induced variables offer high discriminative power to distinguish NSR from AF. We showed that the observation time can be dramatically shortened without significant loss of accuracy using a two-variable model. Finally, we documented the robustness of the proposed *γ*-analysis for noisy data.

The proposed *γ*-analysis quantifies how the selected induced variables enable better use of the information contained in the observed variables. The positioning of the proposed technique in the automatic decision process (depicted in Fig. 1) is a preliminary step to select a limited number of relevant variables before application of any classification technique: the *γ*-analysis is a descriptive tool, not a decision tool. Due to the so-called “curse of dimensionality” that arises when a large number of variables are used as the input of a given classifier, one cannot use all of the available variables. Here, we narrowed our study to a limited number of operators (mean and standard deviation), but one can decide to use all of the well-known variables frequently used in heart rate variability (HRV) analysis such as the SDSD, RMSSD, pNN50, LF/HF power ratio, SD1, SD2, and approximate entropy. Combined with an arbitrary order of time derivation, this can lead to a dramatically large number of variables. To avoid overfitting or non-reasonable training time, one should limit the number of variables used as inputs of a classifier. This is what the proposed γ-metric is used for. The potential advantages are twofold: potentially better classification results (overfitting issue) and lower computational and resource loads required to train the classifier and to process the embedded real-time classification. *γ*-analysis is complementary to other well-known techniques such as Principal Component Analysis (PCA)^25^ and Multiple Discriminant Analysis (MDA)^26^ for variable selection. However, whereas PCA attempts to reduce the number of variables through correlation analysis and MDA attempts to find the best linear combinations of variables for discriminating two or more groups, the *γ*-analysis selects which variables of an initial set extract the information with the highest discriminative power. As in the *γ*-analysis, the PCA and MDA techniques help the subsequent classifier to avoid overfitting by reducing the number of input variables. However, as the components of the PCA or the factor for the MDA are a linear combination of the original available variables, all of the original available variables must be processed, stored and combined, which is not desirable in real-time embedded devices. The *γ*-analysis can also be combined with dimension reduction techniques such as PCA and MDA. Moreover, using the covariance matrix to define the ellipsoids, the proposed technique automatically filters out the outliers. As a result, other combinations of variables than the one selected by the *γ*-analysis may have better classification accuracy on a specific training or validation database but lack classification accuracy when applied to previously unseen data. Furthermore, for computational simplicity, the γ-metric evaluates the distance between two datasets as the distance between the surfaces of the modelled ellipsoids along their mean-mean axis (Fig. 2a and b). This approach requires fewer computations than processing the intersection volume and allows for selection from several non-intersecting ellipsoids, unlike the volume-based approach.

For a state characterization problem, particularly in the case of complex systems, the relations among the variables may be more important than the variables themselves. For example, for obesity detection, an induced variable such as the body mass index (BMI) would be expected to yield better classification results than height and weight, even when used jointly^27^. Broadly speaking, induced variables do not generate information but rather enable observation of the available information from another point of view. The induced variables used here for heart rhythm classification were time derivatives. Derivatives of variables with respect to other variables measure how they are locally related and express how a change in one variable affects another variable. Because heart rhythm results from several interacting processes, it exhibits non-linear behaviour^28^. Time derivatives of heart rate or heart period appear to be adapted to catch heart rhythm dynamics that explore accelerations and jerks of the heart rate through the first and second time derivative of RR interval time series. Notably, this approach appears to be very innovative because although several techniques are listed by the Heart Rate Variability Task Force^14^, no similar approach is mentioned.

Because AF can have dramatic consequences, recommendations promote mass screening of AF^1^, encouraging industry to actively develop hardware and software devices devoted to automatic AF detection. Because the trend is toward mobile devices that must be compact and low-power, there is a need for a rapid, accurate method of studying short physiological (i.e., RR interval or equivalent) time series to reach real-time automatic medical monitoring (e-Health). Here, we show that it is possible to optimize the use of the time series’ informational content. We outline and validate a methodology that enables study and discrimination of observed variable dynamics. The soundly computed new time series allow for dramatically reducing the observation time without significant loss of accuracy. We illustrate the usefulness of the methodology by investigating the accuracy of an automatic basic heart rhythm classifier, but there are other similar potential applications in the medical domain. At the individual scale, other physiological signals than ECG could be considered such as EEG, providing new approaches to explore the dynamics of brain electrical activity during epileptic seizures. Time series of various successive discrete measures (self-monitored blood glucose concentrations or blood concentrations of tumour markers) could be used for earlier detection of poorly controlled diabetes or cancer recurrence.

## Methods

### Time series extraction

The time series studied in this paper were obtained from observations of instantaneous heart cycle periods and consisted of successive RR intervals. They were extracted from the online PhysioNet^29^ website, from the open MIT-BIH Normal Sinus Rhythm (NSR) Database (nsrdb), the MIT-BIH Normal Sinus Rhythm (NSR) RR Interval Database (nsr2db), and the MIT-BIH Atrial Fibrillation (AF) Database (afdb). The length of the time series was set to correspond to heart rhythm observations spanning 1 min. To obtain the illustrative results depicted in Fig. 2c–f, 20 time series from the NSR group and 20 time series from the AF group were randomly extracted from the nsrdb and afdb databases, respectively. To ensure heart rate overlap between NSR and AF group subsets, the mean heart rates of the randomly selected samples were filtered to obtain 10 subjects in each group (NSR and AF) subset with an average heart rate of 80–100 bpm and 10 subjects in each group (sinus tachycardia and AF) subset with an average heart rate of 100–120 bpm. For simplicity, regardless of the underlying heart rate (normal [80–100 bpm] or increased heart rate [tachycardia, 100–120 bpm]), NSR is used in the remainder of the paper as a generic term to differentiate sinus rhythm from atrial fibrillation. PhysioNet describes the selected databases as including “beat annotation files for 54 long-term ECG recordings of subjects in normal sinus rhythm (30 men, aged 28.5 to 76, and 24 women, aged 58 to 73) and 18 long-term ECG recordings of subjects having no significant arrhythmias (5 men, aged 26 to 45, and 13 women, aged 20 to 50)”. Accordingly, the sinus tachycardia sequences randomly extracted from these databases likely correspond to common daily increased heart rate associated with exercise, REM sleep, anxiety, or coffee drinking, as they are not specifically specified by PhysioNet annotations. To obtain the NSR and AF comparison results depicted in Figs 2 and 4, larger subsets were randomly extracted from the nsrdb, nsr2db and afdb databases. In total, 4,500 and 2,900 1-min RR intervals were collected and used for the NSR and AF groups, respectively.

Although PhysioNet is an extremely rich library of physiological databases that are extensively used for academic and industrial purposes, it is difficult to master the data it contains because they are retrospective and the available information about clinical and acquisition conditions is at least partially deficient. The number of subjects that constitute the databases is limited, and their representativeness is questionable. Our preliminary analysis showed that in the NSR group, the variability of the RR interval of each of the 1-min sequences varied from values that were very different from those of the AF group to values that were very close to or overlapping with the AF group. In contrast, in the AF group, some of the 1-min RR interval sequences were more regular than others and some were sufficiently regular to be similar to those in the NSR group. Inter-individual variability is high in biological and medical problems, particularly in heart rhythm dynamics, and thus, we cannot be certain that NSR and AF data files available from PhysioNet are sufficiently representative of their rhythm categories for our purposes. To minimize this limitation, we randomly selected many 1-min RR interval sequences after pooling and shuffling, which introduced both intra- and inter-individual variability.

### Induced Time Series Generation

Time derivatives of the time series were obtained using the forward finite-difference formula, defined as

1 $$\frac{dx}{dt}[n]\approx \frac{x[n+1]-x[n]}{{t}_{n+1}-{t}_{n}},$$

but similar results are expected when the backward and central difference formulas are used. We studied the time series derivatives up to the 10^th^ order. According to formula (1), induced time series should be correlated and, as correlated variables may not be useful in a classification problem, the correlations between the generated variables were investigated. The correlation matrix was obtained by evaluating the correlations between the i^th^- and j^th^-order derivatives of the initial RR interval time series of 20 distinct randomly selected 1-min RR interval time series from the NSR group and 20 distinct randomly selected 1-min RR interval time series from the AF group. The resulting absolute values of the Pearson correlation coefficients are represented in line *i* and column *j* by means of a specific colour map.

### Time series analysis

The results of 2 mathematical operators applied to each extracted and induced time series were used to characterize each time series. For simplicity, these operators were limited to the arithmetic mean and the unbiased sample variance but could have been any of the typical time, frequency, or informational domain operators^14^. A two-sided unpaired t-test was used to compare the means of the means and standard deviations of the RR interval time series and their first ten time derivatives for 4,500 samples (NSR group) and 2,900 samples (AF group). The p-values of the differences are provided in Table 1, and p < 0.05 was considered statistically significant.

### Discriminant variable selection

Contrary to the Principal Component Analysis (PCA)^25^ that generates a set of linearly uncorrelated variables from the initial variables or the Multiple Discriminant Analysis (MDA)^26^ that reduces the number of variables by finding linear combinations of the initial variables and selects only the most discriminant, the selection of the relevant variables from all of the potentially discriminant state variables computed (i.e., mean or standard deviation of the original time series or of one of the first ten time derivatives) is performed using a new metric that we proposed called the *γ*-metric. The *γ*-metric is defined as

2 $${\gamma }_{M}=\sum _{k1=1}^{K}\,\sum _{k2 < k1}{d}_{k1k2},$$

where K is the number of groups (in this paper, K = 2 for the AF and NSR groups) and *d*_*k*1*k*2_ is a quantity that is proportional to the algebraic distance between the group denoted *k*_*1*_ and the group denoted *k*_*2*_ along their mean-mean axis (Details on the **γ**-metric are provided in the supplementary information document named Appendix A). This metric is used to select the minimum set of variables that maximizes *γ*_*M*_ in the space defined by the M selected variables. In this space, the groups to discriminate are modelled by multidimensional ellipsoids whose axes are defined by the eigenvectors of the selected variables covariance matrix (see Fig. 2a and b for a graphical representation of the algebraic distance between two ellipsoids). A univariate *γ*-analysis was performed by computing the *γ*-metric value for each potentially discriminant state variable calculated. A multivariate *γ*-analysis was performed by computing the *γ*-metric value for all n-variable (n from 1 up to 22) combinations of the generated variables (means and standard deviations of the RR interval time series and each of the first ten derivatives) as reported in Fig. 4e and Table 2.

### Heart rhythm classification

The unregularized logistic regression was arbitrarily chosen to separate the study groups (NSR and AF). The classification accuracy was obtained using this classifier in a univariate and multivariate mode. In the univariate mode, the classifier input variable was the result of applying the mean or standard deviation operator to the 1-min RR interval time series or to one of its first ten time derivatives (resulting in 22 different state variables). In the multivariate mode, the classifier input was a combination of 1 to 22 of these computed state variables. For example, in the case of the highest *γ*_2_ or highest *γ*_3_ in Fig. 5, only the operator-and-variable combinations summarized in Table 2 were considered. To emphasize the interest of using the *γ*-value to select relevant inputs of the classification model, we studied the classification accuracy of all pairs of the 22 variables. To analyse the logistic regression accuracy according to the length of the observed time series, we challenged the classifier with time series corresponding to lengths of 1, 2, 3, 4, 5, 6, 7, 8, 9, 10, 15, 20, 25, 30, 40 and 50 s that were generated from the initial 1-min RR interval time series.

All of the logistic regression accuracy results were obtained using a dataset of RR interval time series partitioned into a training subset (80% of the total dataset) and a validation subset (20% of the total dataset) using the *Classification Learner tool* from the MATLAB^30^ *Statistics and Machine Learning Toolbox*.

### RR interval perturbations

To test the robustness of the proposed technique, several sources of perturbations were added to the original AF and NSR databases. These perturbations were added to reflect the typical sources of noise or artefacts that are commonly observed in embedded devices. First, timing uncertainty (TU), reflecting the accuracy of the R peak positioning, was represented as uniformly distributed random variables on each R peak. Such randomly distributed variables were generated in a [−*T*, *T*] interval where *T* ∈ {1, 2, 3, 4, 5, 10, 15, 20, 30, 40, 50, 100} ms. Moreover, R peak detectors sometimes suffer from missing detection due to weak beat amplitudes. To model this phenomenon and the signal disruption, a percentage of missing beats (MB) was added to the initial databases. This percentage relates to the total number of beats for a given sample of the database. If the length of the considered samples was *L*, (*u* · *L*) indexes were randomly selected and the corresponding beats were discarded. The indexes were uniformly distributed in [1, *L*], and the value of *u* was taken in {1%, 2%, 3%, 4%, 5%, 10%, 15%, 20%, 30%}. Finally, premature cardiac contractions were also modelled as they may interfere with classification of heart rhythm, especially when AF is considered. Premature atrial contractions (PAC) and premature ventricular contractions (PVC) were modelled with the same percentage technique as that used for the missing beats. The model used for PAC is the insertion of a new beat $\tilde{b}$ between two original beats indexed by *i* and *i* + 1 and denoted by *b*_*i*_ and *b*_*i*+1_, respectively. $\tilde{b}$ was inserted at least 200 ms after *b*_*i*_ and 200 ms before *b*_*i*+1_, and *b*_*i*+1_ and the subsequent beats were shifted by $|\tilde{b}-{b}_{i}|$. The model used for PVC is the insertion of a new beat $\tilde{b}$ between two original beats *b*_*i*_ and *b*_*i*+1_. In this case, $\tilde{b}$ was inserted at least 200 ms after *b*_*i*_ and 200 ms before *b*_*i*+1_, and *b*_*i*+1_ was deleted. Although the NSR samples were corrupted by TU, MB, PAC and PVC, the AF samples were only corrupted by TU and MB.

Pooled and noisy databases were formed by merging 25% of original NSR samples, 25% of NSR samples with MU, 25% of NSR samples with PAC and 25% of NSR samples with PVC. The MU percentages used were uniformly chosen from {1%, 2%, 3%, 4%, 5%, 10%, 15%, 20%}, and the PAC and PVC percentages were uniformly chosen from {1%, 2%, 3%, 4%, 5%, 10%, 15%, 20%, 30%}.

## Electronic supplementary material

Supplementary PDF File
