# Supplementary material for: Heart rhythm characterization through induced physiological variables
Source: Sci Rep. 2017 Jul 11;7:5059. doi: 10.1038/s41598-017-04998-7 (PMC5505978; doi:10.1038/s41598-017-04998-7)
Supplement: Supplementary file 1 — Supplementary PDF File [file 41598_2017_4998_MOESM1_ESM.pdf]

Title:

Heart rhythm characterization through induced physiological variables

Authors

Jean-François Pons[1*], Zouhair Haddi[2], Jean-Claude Deharo[3,4], Ahmed Charaï[1], Rachid Bouchakour[1], Mustapha Ouladsine[2], Stéphane Delliaux[3,5]

Acknowledgements

This work, which is part of the APPRISE/HIT project, was performed with the support of the A*MIDEX project (n° ANR-11-IDEX-0001-02) funded by the «Investissements d'Avenir» program of the French Government, which is managed by the French National Research Agency (ANR).

Author Contributions

S. D. conceptualized the theoretical and applied problems, designed the study, performed the exploratory data processing, analyzed the results, wrote the paper and led the research. J.-F. P. conceptualized the theoretical problem and its mathematical solutions, developed the γ-metric concept and performed its mathematical development, performed extensive data processing and the γ-analysis, analyzed the results and wrote the paper. Z. H. extracted the time series from the databases and performed preliminary data processing and preliminary classification studies. J.-C. D. formulated the medical problem and additional constraints. M. O. advised on the data-processing methods. All authors, including R. B. and A. C., discussed the results and commented on the manuscript.

Author Information

The authors declare no competing financial interests. Correspondence and requests for materials should be addressed to stephane.delliaux@univ-amu.fr and jean-francois.pons@im2np.fr

Author affiliations

[1] Aix Marseille Univ., Univ. Toulon, CNRS, IM2NP, Marseille, France

[2] Aix Marseille Univ., Univ. Toulon, CNRS, ENSAM, LSIS, Marseille, France

[3] Aix Marseille Univ., IRBA, DS-ACI, Marseille, France

[4] APHM, Hôpital La Timone, Service de Cardiologie du pôle cardiovasculaire et thoracique, Marseille, France

[5] APHM, Hôpital Nord, Service des Explorations Fonctionnelles Respiratoires, Pôle cardiovasculaire, Marseille, France

Corresponding author

Jean-François Pons
IM2NP / Polytech'Marseille
5 rue Enrico Fermi Bâtiment NEEL
Technopôle de Château Gombert
13453 MARSEILLE Cedex 13
France
Phone : +33413554024

Subject codes

Atrial Fibrillation, Computational Biology, Information Technology

Appendix A. Details of the γ-metric

The details of the γ-metric expression described in (2) are given here. Let us assume that the state of the observed system is initially defined by M state variables $\{Y_m\}_{m\in\{1..M\}}$ and that we are interested in finding an induced variable Y_{M+1} to better discriminate the different possible states. The system of interest is also assumed to be in one of K possible states denoted by $k \in \{1..K\}$. Starting with the M original variables, each of the K possible states is characterized by a covariance matrix denoted by

$$W_{k,M} = cov(Y \in k). \tag{A1}$$

$W_{k,M}$ is an M x M symmetric positive semi-definite matrix, its eigenvalues $\{\lambda_i\}_{i\epsilon\{1..M\}}$ are positive ($\forall i \in \{1..M\}, \lambda_i \geq 0$), and $W_{k,M}$ is diagonalizable. The normalized eigenvectors of $W_{k,M}$ associated with the eigenvalues $\{\lambda_{k,i}\}_{i\epsilon\{1..M\}}$ are denoted $\{\overrightarrow{u_{k,i}}\}_{i\epsilon\{1..M\}}$. These vectors associated with their eigenvalues allow for determination of the principal axis of the point distribution for a given state k, for example, in the principal component analysis (PCA)[25]. These vectors can also be viewed as the direction of the M semi-principal axes of length $\{\lambda_{k,i}\}_{i\epsilon\{1..M\}}$ of an M-dimension ellipsoid centred on the M-dimension mean $\overrightarrow{\mu_k}$ representing the distribution of the observed elements in state k. Consequently, to determine whether the K states are well discriminated using the M variables, we propose the use of a metric representative of the algebraic distance between these K ellipsoids.

The expression of the γ-metric in (2) represents the sum of the algebraic distance between the ellipsoids along the mean-mean axis (given by $\overrightarrow{\mu_{k1}\mu_{k2}}$) for each couple of states among the K possible states. The value of d_{k1k2} is defined by

$$d_{k1k2} = \frac{1}{\alpha_{k1k2}}\left(\|\overrightarrow{\mu_{k1}\mu_{k2}}\| - \left(d_{k1,k1k2} + d_{k2,k1k2}\right)\right), \quad \forall k1 \in \{1..K\}, \forall k2 \in \{1..K\}, k1 < k2, \tag{A2}$$

where μ_{k1} is the M-dimension mean of the state $k1$, μ_{k2} is the M-dimension mean of the state $k2$, and α_{k1k2} is a normalization factor defined by

$$\alpha_{k1k2} = \sqrt{\sum_{m=1}^{M} \lambda_{k1,m}} + \sqrt{\sum_{m=1}^{M} \lambda_{k2,m}} \tag{A3}$$

and $d_{k1,k1k2}$ and $d_{k2,k1k2}$ are defined by

$$d_{k1,k1k2} = \frac{1}{\sqrt{\sum_{m=1}^{M} \frac{\tilde{\mu}_{k1,m}^2}{\lambda_{k1,m}^2}}} \tag{A4}$$

and

$$d_{k2,k1k2} = \frac{1}{\sqrt{\sum_{m=1}^{M} \frac{\tilde{\mu}_{k2,m}^2}{\lambda_{k2,m}^2}}}, \tag{A5}$$

where $\tilde{\mu}_{k1,m}^2$ and $\tilde{\mu}_{k2,m}^2$ represent the coordinates of the normalized mean-mean vector

$$\overrightarrow{\mu_{k1k2}} = \frac{\overrightarrow{\mu_{k1}\mu_{k2}}}{||\overrightarrow{\mu_{k1}\mu_{k2}}||} \tag{A6}$$

expressed in the orthogonal basis formed by the eigenvectors of the $k1^{th}$ ellipsoid and in the orthogonal basis formed by the eigenvectors of the $k2^{th}$ ellipsoid, respectively.

As a result, the criterion used to determine if a new variable helps discriminate the different states is given as follows

$$\gamma_{M+1} > \gamma_M, \tag{A7}$$

and an induced variable is selected only if this condition is true.
